# Incidence of Cracked Teeth Before, During, and After the Covid‐19 Pandemic—A Retrospective Analysis in a German Private General Practice

**DOI:** 10.1002/cre2.70239

**Published:** 2025-10-07

**Authors:** Jörg Philipp Tchorz, Patrizia Ladar, Maria Lessani, Sebastian Fitzek

**Affiliations:** ^1^ Division for Endodontics, Center for Operative Dentistry and Periodontology, Department of Dentistry, Faculty of Medicine and Dentistry Danube Private University Krems Austria; ^2^ Endoclinic Practice limited to Endodontics North London UK; ^3^ Health Services Research Group, Medical Images Analysis and Artificial Intelligence Danube Private University Krems Austria

**Keywords:** Covid‐19, cracked teeth, cusp fracture, split tooth

## Abstract

**Objectives:**

The Covid‐19 pandemic increased stress, which may have led to more bruxism, TMDs, and thus tooth fractures. While previous studies, primarily from specialized clinics, reported an increase in cracked teeth during the pandemic, this retrospective study aimed to assess whether similar trends were observed in a general dental practice in Bavaria, Germany, which remained fully operational throughout the pandemic.

**Material and Methods:**

Patient records from 2018 to 2023 (*n* = 4709 patients, 10,018 visits) were analyzed for symptomatic tooth fractures, categorized as wall/cusp fractures, incomplete cracks, or complete fractures.

**Results:**

Statistical analysis using the Mann–Whitney *U* test revealed no significant differences in fracture incidence between pre‐pandemic (2018–2019), pandemic (2020–2021), and post‐pandemic (2022–2023) periods. Mandibular molars were the most frequently affected teeth (7.94%–12.43%), and composite restorations were most associated with fractures (51.85%).

**Conclusions:**

Contrary to previous findings from endodontic practices, this study found no pandemic‐related surge in tooth fractures, suggesting that referral bias or patient selection in specialized settings may influence reported trends.

## 1 Introduction

It has been reported that the Covid‐19 pandemic has led to a significant rise in psychological distress (Pfefferbaum and North 2020; Brooks et al. 2020). The mental health challenges have not only affected individuals' well‐being but have also reported cascading effects on dental health, for example, by an increase in the prevalence of bruxism and temporomandibular disorders (TMDs) (Mirhashemi et al. 2022; Winocur‐Arias et al. 2022; Shalev‐Antsel et al. 2023). Bruxism has been defined as a repetitive jaw‐muscle activity characterized by clenching or grinding of the teeth, and/or by bracing or thrusting of the mandible, and is often linked to psychological factors, such as stress and anxiety (Lobbezoo et al. 2013; Wieckiewicz et al. 2014; Przystańska et al. 2019). The occlusal force, caused by bruxism, can lead to dental complications such as tooth sensitivity, cracked teeth, and consequential pulpal and periapical disease (Koyano et al. 2008; Manfredini et al. 2013; Gund et al. 2022; L. T. Thayer and Ali 2022). Cracks in teeth can manifest in various forms, ranging from superficial craze lines in the enamel to more severe fractures that even extend into the dentin or pulp (Patel et al. 2025). Common clinical appearances are fractured cusps, where a portion of the tooth's chewing surface breaks off; cracked teeth, which involve an incomplete crack with occlusal origin; and split teeth, where the tooth is divided into distinct segments (Patel et al. 2025).

In the past few years, several authors described a causal relationship between the stress induced by the Covid‐19 pandemic, a subsequent observed rise in parafunctional habits like bruxism, and the resulting increase in fractured teeth (Winocur‐Arias et al. 2022; Shalev‐Antsel et al. 2023; Nosrat et al. 2022; Popescu et al. 2024; Abdellatif et al. 2024). While this proposed etiology is clinically plausible, it is crucial to interpret these findings with caution. The majority of published data supporting this trend originate from specialized clinics and endodontic practices. These settings inherently attract a patient population seeking care for complex dental pain, potentially leading to a significant selection bias where both the prevalence of bruxism and its consequences, such as tooth cracks and fractures, are overrepresented. This bias was likely exacerbated during pandemic lockdowns, as many general dental practitioners reduced services, further referring only the most acute cases—including symptomatic cracks and fractures—to specialists. Consequently, while the stress–bruxism–fracture pathway is theoretically sound, the available evidence may overstate the magnitude of this effect within the general population. Accordingly, this study aims to determine whether similar observations (H_0_) can be made in a general dental practice, which stayed open during the entire pandemic and lockdown period, by analyzing data from the years 2018 to 2023.

## 2 Materials and Methods

This retrospective analysis examined patient records from a single general dental practice in Bavaria, Germany, which remained operational throughout the Covid‐19 pandemic. The protocol was reviewed and approved by the Ethics Committee of the Bavarian State Chamber of Physicians (reference number: 2022‐1108).

The present study employed a retrospective observational design with the objective of analyzing all available cases within a defined population and time period. As such, a formal a priori sample size calculation was not performed. The study aimed for a comprehensive census rather than a sample, as all patients who met the inclusion criteria during the observation period (January 2018 to December 2023) were included in the analysis. This approach ensures a complete representation of the cohort and minimizes selection bias for the given timeframe and setting. The resulting sample size is therefore a function of the actual clinical population and incidence during the study period.

Only patients treated continuously by one practitioner were included. Records were screened for terms such as “crack” and “fracture,” categorized as wall/cusp fractures, incomplete fractures (cracks), or complete fractures (split teeth). Inclusion was limited to symptomatic teeth, defined by a positive percussion or bite test or pronounced sensitivity to cold thermal stimuli. A definitive diagnosis was confirmed through a combination of visual inspection under high magnification with an operating microscope and transillumination. When two different fracture types were diagnosed on the same tooth at the same visit (e.g., wall/cusp fracture with an underlying additional incomplete fracture), both findings were documented. Collected variables included date, fracture type, tooth, restoration type, patient age, and sex. Patients were counted only once per year to prevent multi‐visit bias in the annual total. Descriptive statistics summarized the total patients and incidence rates per fracture type and are presented in Table 1. Given the limited number of years per period (*n* = 2) and the likely non‐normal distribution of incidence rates, a nonparametric Mann–Whitney *U* test was employed to compare pre‐pandemic, pandemic, and post‐pandemic periods, with significance set at 5%. Restoration types were grouped as direct (composite or amalgam), indirect (inlay, partial crown, or crown), or others (no restoration or undefined). An exploratory *χ* ^2^ test assessed whether fracture type distribution varied significantly across restoration categories. Analyses were conducted using Python 3.9.6.

**Table 1:** Number of patients and number of different types of fractures recorded in the years 2018–2023.

Year	Total patients	Wall/cusp fractures	Incomplete fractures (cracks)	Complete fractures	Period	All fractures combined
2018	1592	14 (0.88%)	16 (1.01%)	25 (1.57%)	Pre‐pandemic	131 (4.08%)
2019	1622	40 (2.47%)	8 (0.49%)	28 (1.73%)		
2020	1392	21 (1.51%)	11 (0.79%)	23 (1.65%)	Pandemic	115 (3.91%)
2021	1551	21 (1.35%)	14 (0.9%)	25 (1.61%)		
2022	1925	23 (1.2%)	17 (0.88%)	33 (1.71%)	Post‐pandemic	155 (4.01%)
2023	1936	32 (1.65%)	18 (0.93%)	32 (1.65%)		

## 3 Results

**Figure 1:**
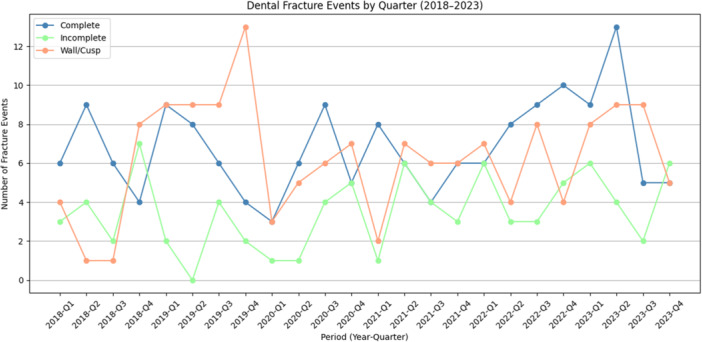
This figure visualizes the number of different fracture events (wall/cusp, incomplete, or complete) broken down by quarter.

Data from 4709 individual patients and 10,018 visits were screened. Total patient visits during the pandemic period (2020–2021) were 8.43% lower than the pre‐pandemic years (2018–2019). During the post‐pandemic period of 2022–2023, the number of patient visits had increased by 20.13% when compared to the pre‐pandemic period. A total of 401 fractures were recorded in 378 teeth between 2018 and 2023. The most common combination, observed in 23 cases where a single tooth had multiple fracture types, was a wall/cusp fracture with an underlying incomplete fracture. No patient was recorded with more than one affected tooth within the same 2‐year period. The gender distribution of the patients indicated that 189 were female and 189 were male, with a mean age of 53.92 years. Pre‐existing restorations were present in 96.03% of fractured teeth (363 of 378), and only 15 teeth (3.97%) were unrestored. The most frequent restoration type was composite (51.85%, *n* = 196), followed by amalgam (23.55%, *n* = 89) and full crowns (11.64%, *n* = 44). The most frequent tooth type fractured were mandibular molars (7.94%–12.43%), followed by maxillary molars (3.70%–9.52%) and maxillary premolars (3.70%–6.08%). Taking all fracture events into account, 24.87% of the teeth had root canal fillings. The percentage of root‐filled teeth in the complete fracture group was only slightly higher (35.54%). A pronounced peak of wall/cusp fractures was observed in 2019 (24.66 per 1000), with lower rates in the other years (Figure 1). The lowest incidence of incomplete fractures was observed in 2019 (4.93 per 1000), with higher rates in other years. The incidence rates of complete fractures ranged from approximately 15.70 to 17.26 per 1000 patients. Mann–Whitney *U* tests revealed no statistically significant differences (*p* = 1.000) between pre‐pandemic and pandemic periods.

**Figure 2:**
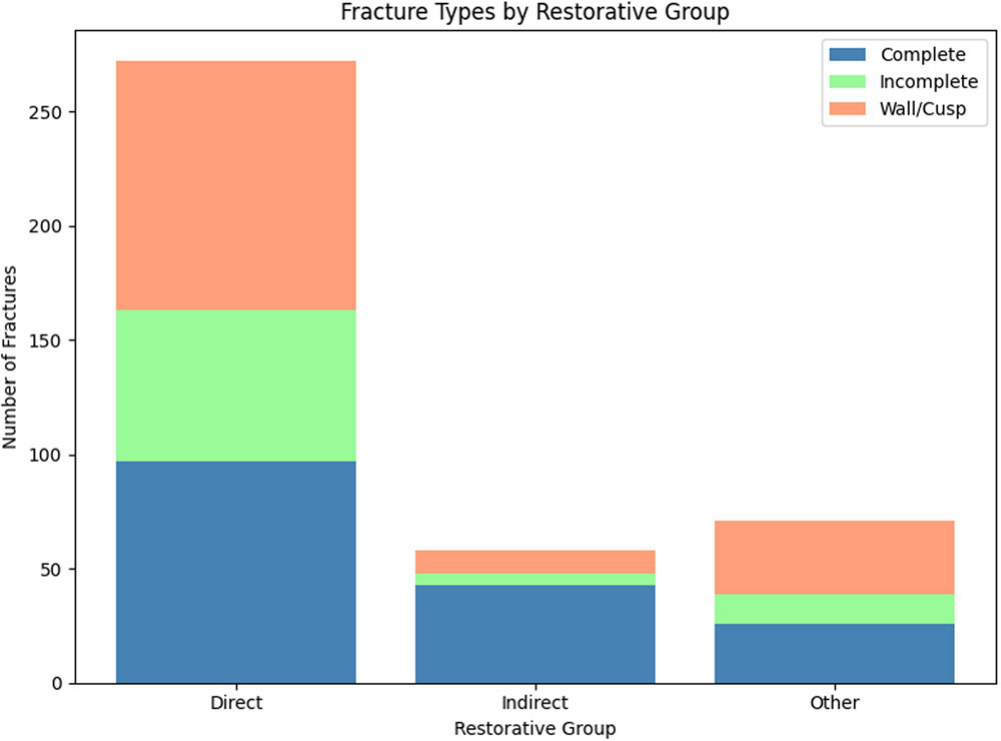
Types of fractures more common within each restorative group. For instance, if the bar for a certain type of fracture is higher, it might suggest a higher prevalence of fractures—but this does not confirm a higher risk per se, only that among fractured teeth, direct restorations, for example, are more frequently observed. The figure represents pooled data for the entire observational period.

Since our dataset primarily contained fractured teeth, a *χ* ^2^ test of fracture versus no fracture was not feasible because entries without fractures were absent. Figure 2 visualizes which types of fractures were more common within each restorative group.

## 4 Discussion

Previous studies have shown the impact of the Covid‐19 pandemic on several aspects of oral health. Winocur‐Arias et al. evaluated the effect on the prevalence of bruxism, oral parafunctions, and painful TMDs and observed a significant increase in parafunction activity in both men and women (Nosrat et al. 2022). Shalev‐Antsel et al. compared the incidence of TMD and bruxism before, during, and after the pandemic and observed a three‐ to four‐fold increase during that time (Shalev‐Antsel et al. 2023). Abdellatif et al. carried out an observational study to analyze the correlation between the stress caused by the Covid‐19 pandemic and parafunctions of the temporomandibular joint and masticatory muscles (Abdellatif et al. 2024). Their major objective was to evaluate whether an increase in stress levels may have led to a higher incidence of fractures and dental injuries. A questionnaire was addressed to all dentists in the Campania Region in Italy, whereby 730 completed surveys were returned, revealing that 61.7% of the dentists stated that the number of patients reporting muscle and joint pain in the morning had increased, 56.7% dentists noted an increase in parafunction, and 53.6% an increase in dental fractures during the pandemic period. However, as this study was based on the subjective perception of the respondents, direct conclusions cannot be drawn. Although the author of the present study also subjectively perceived an increased fracture rate during the pandemic, this observation was not confirmed.

In comparing the results of the present study with others, it was observed that a significant increase in cracks and/or fractures was evident in clinics and specialized endodontic practices. Accordingly, higher incidences may be due to referral bias or a higher‐risk patient population. Nosrat et al. evaluated the rate of cracks and other etiologic factors from 2019 until 2021 in two endodontists' practices (2022). They assessed the etiology of endodontic complaints in 2440 teeth and observed a significant peak in the incidence of cracks in 2020, followed by a decrease in the following year. The average age of patients with cracked teeth ranged between 40 and 60 years and was comparable to observations from this study. Ek et al. investigated the prevalence of vertical root fractures (VRFs) in a university endodontic program and a private endodontics practice and reported an overall incidence of 2.01%, further reporting that there was an increased rate during the Covid‐19 period (Ek et al. 2023). The percentage in the private endodontic practice (2.62%) was slightly higher compared to the university (1.80%). The limitations of the study included the fact that the prevalence was evaluated via treatment notes from different observers. Although the same search terms were used for evaluation purposes in both facilities, it cannot be said with certainty that all practitioners used exactly the same terminology in their documentation. Therefore, in this study, it was decided to evaluate the patient data collected by only one dental practitioner to reduce any documentary‐dependent bias. The decision to limit inclusion to symptomatic teeth was a necessary methodological choice to establish a clear temporal relationship between the clinical finding and the study period. As asymptomatic cracks are often incidental findings of indeterminate age, their inclusion would have introduced significant uncertainty regarding whether the fracture actually occurred during the defined observational timeframe. While this approach ensures temporal accuracy for the analyzed cases, it consequently excludes the subpopulation of teeth with asymptomatic cracks, which may represent an earlier stage in the natural history of the condition. Although clinical visual diagnosis of dental cracks is susceptible to observer bias, it was shown that transillumination and high magnification result in high accuracy and high sensitivity (Kindaro et al. 2025).

Furthermore, the results of single‐center observational studies depend heavily on the patients' socioeconomic context, where factors like insurance coverage may influence the outcomes. Our study was conducted in a high‐income area within Germany, a country with a robust universal healthcare system that includes high rates of dental insurance coverage. This specific context likely contributed to a lower observed incidence and severity of fractures, as improved access to care enables preventive measures and early management of risk factors like caries. Another possible explanation for higher fracture rates observed by others may be that there were simply fewer patient visits during the respective period, for example, during the lockdown (Popescu et al. 2024; Guo et al. 2022). A lower number of patient visits and a constant number of symptomatic cracks and fractures, classified as emergencies, may distort the calculation of incidence rates accordingly. Guo et al. examined changes in the number of patient visits and types of oral services in an oral emergency department throughout the pandemic and observed decreased daily visits up to 50% from 314 to 155 patients per day (Guo et al. 2022).

In this study, the data analysis was carried out based on data from a general dental practice that was fully operational during the entire pandemic years, including the lockdown period, and dental services were not limited to emergencies. The number of patients was 14.2% lower in 2020 and reduced by 4.4% in 2021. In the following year, 2022, the number of patients was 18.7% higher compared to the pre‐pandemic year 2019. This increase is certainly due to the catch‐up effect of patients who did not visit a dentist during the pandemic. We did not take the total visits for incidence calculation, but the number of patients per year. If the number of visits is used as a basis, then treatments involving several necessary visits (e.g., prosthodontic procedures) automatically lead to a distortion of data and an overestimation. Although we recorded whether a patient had multiple fractures within the same year or the same period (pre‐pandemic, pandemic, or post‐pandemic), our pseudo‐anonymized data did not support tracking patients across multiple years to identify recurrent fractures over time.

With the present outcome, the null hypothesis (H0) was rejected. Despite prior assumptions, the incidence of different types of tooth fractures remained stable over the observational period, emphasizing that pandemic‐related stress, as a possible trigger for bruxism, was not a primary driver of a higher fracture rate.

## Author Contributions

**Jörg Philipp Tchorz:** data collection, methodology, interpretation, original draft. **Patrizia Ladar:** data collection, writing – review and editing. **Maria Lessani:** conceptualization, writing – review and editing. **Sebastian Fitzek:** data analysis, interpretation, writing – review and editing.

## Ethics Statement

This study was conducted in accordance with the ethical standards of the institutional and national research committee and with the Helsinki Declaration and its later amendments or comparable ethical standards. The protocol was reviewed and approved by the Ethics Committee of the Bavarian State Chamber of Physicians (reference number: 2022‐1108).

## Conflicts of Interest

The authors declare no conflicts of interest.

## Data Availability

The data that support the findings of this study are available upon request from the corresponding author.

## References

[cre270239-bib-0001] Abdellatif, D. , A. Iandolo , M. Pisano , R. Fornara , G. Sangiovanni , and M. Amato . 2024. “The Incidence of Dental Fractures in the Italian Population During the COVID‐19 Pandemic: An Observational Study.” Journal of Conservative Dentistry and Endodontics 27: 146–153. 38463480 10.4103/JCDE.JCDE_241_23 PMC10923222

[cre270239-bib-0002] Brooks, S. K. , R. K. Webster , L. E. Smith , et al. 2020. “The Psychological Impact of Quarantine and How to Reduce It: Rapid Review of the Evidence.” Lancet 395: 912–920. 32112714 10.1016/S0140-6736(20)30460-8 PMC7158942

[cre270239-bib-0003] Ek, B. , S. Zweig , R. G. Roges , et al. 2023. “Prevalence of Vertical Root Fractures in a University Endodontics Program Versus a Private Endodontics Office.” International Journal of Dentistry 2023: 2098629. 38149084 10.1155/2023/2098629 PMC10751159

[cre270239-bib-0004] Gund, M. P. , K. T. Wrbas , M. Hannig , and S. Rupf . 2022. “Apical Periodontitis After Intense Bruxism.” BMC Oral Health 22: 91. 35331220 10.1186/s12903-022-02123-3 PMC8951715

[cre270239-bib-0005] Guo, H. Q. , T. Xu , J. Pan , A. P. Ji , M. W. Huang , and J. Bai . 2022. “A Retrospective Study of Oral Emergency Services During COVID‐19.” International Dental Journal 72: 236–241. 34785063 10.1016/j.identj.2021.09.004 PMC8483898

[cre270239-bib-0006] Kindaro, V. , H. Molland , S. Shirbegi , P. Renner , and U. Krishnan . 2025. “Diagnostic Accuracy of Methods Used to Detect Cracked Teeth.” Clinical and Experimental Dental Research 11: e70138. 40304312 10.1002/cre2.70138 PMC12042108

[cre270239-bib-0007] Koyano, K. , Y. Tsukiyama , R. Ichiki , and T. Kuwata . 2008. “Assessment of Bruxism in the Clinic.” Journal of Oral Rehabilitation 35: 495–508. 18557916 10.1111/j.1365-2842.2008.01880.x

[cre270239-bib-0008] Lobbezoo, F. , J. Ahlberg , A. G. Glaros , et al. 2013. “Bruxism Defined and Graded: An International Consensus.” Journal of Oral Rehabilitation 40: 2–4. 23121262 10.1111/joor.12011

[cre270239-bib-0009] L. T. Thayer, M. , and R. Ali . 2022. “The Dental Demolition Derby: Bruxism and Its Impact—Part 1: Background.” British Dental Journal 232: 515–521. 35459823 10.1038/s41415-022-4143-8 PMC9033581

[cre270239-bib-0010] Manfredini, D. , E. Winocur , L. Guarda‐Nardini , D. Paesani , and F. Lobbezoo . 2013. “Epidemiology of Bruxism in Adults: A Systematic Review of the Literature.” Journal of Orofacial Pain 27: 99–110. 23630682 10.11607/jop.921

[cre270239-bib-0011] Mirhashemi, A. , M. R. Khami , M. Kharazifard , and R. Bahrami . 2022. “The Evaluation of the Relationship Between Oral Habits Prevalence and COVID‐19 Pandemic in Adults and Adolescents: A Systematic Review.” Frontiers in Public Health 10: 860185. 35359778 10.3389/fpubh.2022.860185 PMC8963731

[cre270239-bib-0012] Nosrat, A. , P. Yu , P. Verma , O. Dianat , D. Wu , and A. F. Fouad . 2022. “Was the Coronavirus Disease 2019 Pandemic Associated With an Increased Rate of Cracked Teeth?” Journal of Endodontics 48: 1241–1247. 35835260 10.1016/j.joen.2022.07.002 PMC9273286

[cre270239-bib-0013] Patel, S. , P. H. Teng , W. C. Liao , et al. 2025. “Position Statement on Longitudinal Cracks and Fractures of Teeth.” International Endodontic Journal 58: 379–390. 39840523 10.1111/iej.14186 PMC11812625

[cre270239-bib-0014] Pfefferbaum, B. , and C. S. North . 2020. “Mental Health and the Covid‐19 Pandemic.” New England Journal of Medicine 383: 510–512. 32283003 10.1056/NEJMp2008017

[cre270239-bib-0015] Popescu, A. M. , O. A. Diaconu , S. M. Popescu , et al. 2024. “Cracked Teeth and Vertical Root Fractures in Pandemic Crisis—Retrospective Study.” Current Health Sciences Journal 50: 237–245. 39371055 10.12865/CHSJ.50.02.09 PMC11447506

[cre270239-bib-0016] Przystańska, A. , A. Jasielska , M. Ziarko , et al. 2019. “Psychosocial Predictors of Bruxism.” BioMed Research International 2019: 2069716. 31737656 10.1155/2019/2069716 PMC6815662

[cre270239-bib-0017] Shalev‐Antsel, T. , O. Winocur‐Arias , P. Friedman‐Rubin , et al. 2023. “The Continuous Adverse Impact of COVID‐19 on Temporomandibular Disorders and Bruxism: Comparison of Pre‐ During‐ and Post‐Pandemic Time Periods.” BMC Oral Health 23: 716. 37794398 10.1186/s12903-023-03447-4 PMC10552226

[cre270239-bib-0018] Wieckiewicz, M. , A. Paradowska‐Stolarz , and W. Wieckiewicz . 2014. “Psychosocial Aspects of Bruxism: The Most Paramount Factor Influencing Teeth Grinding.” BioMed Research International 2014: 469187. 25101282 10.1155/2014/469187 PMC4119714

[cre270239-bib-0019] Winocur‐Arias, O. , E. Winocur , T. Shalev‐Antsel , et al. 2022. “Painful Temporomandibular Disorders, Bruxism and Oral Parafunctions Before and During the COVID‐19 Pandemic Era: A Sex Comparison Among Dental Patients.” Journal of Clinical Medicine 11: 589. 35160041 10.3390/jcm11030589 PMC8837112

